# Xuanfei Formula inhibited RSV infection by normalizing the SREBP2-mediated cholesterol synthesis process

**DOI:** 10.3389/fmicb.2024.1387062

**Published:** 2024-05-02

**Authors:** Huan Qin, Jin Luo, Nan Zhao, Wange Lou, Peng Chen, Huihao Wang, Zishu Pan, Xiaoli Xiong

**Affiliations:** ^1^College of Life Sciences, State Key Laboratory of Virology, Wuhan University, Wuhan, China; ^2^Department of Integrated Chinese and Western Medicine, Wuhan Children’s Hospital (Wuhan Maternal and Child Healthcare Hospital), Tongji Medical College, Huazhong University and Technology, Wuhan, China; ^3^Institute of Maternal and Child Health, Wuhan Children’s Hospital (Wuhan Maternal and Child Healthcare Hospital), Tongji Medical College, Huazhong University and Technology, Wuhan, China; ^4^Department of Respiratory Medicine, Wuhan Children’s Hospital (Wuhan Maternal and Child Healthcare Hospital), Tongji Medical College, Huazhong University and Technology, Wuhan, China; ^5^Information Department, Wuhan Children’s Hospital (Wuhan Maternal and Child Healthcare Hospital), Tongji Medical College, Huazhong University and Technology, Wuhan, China; ^6^Clinical Laboratory, Wuhan Children’s Hospital (Wuhan Maternal and Child Healthcare Hospital), Tongji Medical College, Huazhong University of Science and Technology, Wuhan, China

**Keywords:** RSV, traditional Chinese medicine, cholesterol homeostasis, anti-RSV, metabolism reprograming

## Abstract

**Background and aims:**

Respiratory syncytial virus (RSV) is the major cause of lower respiratory tract infections in children and the elderly, often progressing to pneumonia and severe sequelae. However, there are currently no feasible and cost-effective interventions with proven efficacy for children, making medications with anti-RSV activity urgently needed. Traditional Chinese medicine has shown promising therapeutic efficacy in alleviating viral infection symptoms. Therefore, we aimed to develop effective strategies for RSV treatment based on traditional Chinese medicine.

**Methods and results:**

The infection status was assessed in BALB/c mice with or without Xuanfei Formula (XFF) treatment over a one-week period using H&E staining, cytokine assays and RSV titer testing after RSV challenge. Remarkably, on the first day of XFF intervention, both the pro-inflammation cytokine levels in the serum and RSV-N gene copies in the lung of mice were plummeted, compared to the RSV-infected group. This implied that XFF might possess the immune-independent anti-RSV capability. To elucidate the underlying mechanism, we employed transcriptome analysis followed by k-means analysis. The reversal effects of XFF against RSV primarily focused on the processes of innate and adaptive immunity. Additionally, we found that XFF administration corrected the disordered fatty acid and cholesterol metabolism processes during RSV infection. Lipidomics profiling indicated consistent cholesterol abundance with transcriptional changes but not fatty acids. Cholesterol synthesis-related genes mRNA levels and cholesterol synthesis intermediates detection supported XFF’s repression upon cholesterol biosynthesis. Aberrantly increased cholesterol production has been reported as necessary for RSV infection. To mimic that, we observed lovastatin treatment inhibited RSV replication and pro-inflammation cytokine expression *in vitro*. Transcription factor prediction of differentially expressed genes (DEGs) involved in cholesterol synthesis implicated SREBP2. Through network pharmacology, stigmasterol and β-sitosterol were identified as the effective active ingredients within the XFF, with the help of further molecular docking and mass spectrum detection. *In vitro* experiments demonstrated β-sitosterol and stigmasterol reinforced the bonding between SREBP cleavage-activation protein (SCAP) and insulin-induced gene proteins (INSIGs) to inhibit SREBP2 cleavage maturation and consequent RSV infection.

**Conclusion:**

Xuanfei Formula (XFF) exhibits excellent anti-RSV efficacy by inhibiting SREBP2-mediated cholesterol synthesis to reduce RSV replication and ameliorate inflammation in the lung of infected mice.

## Introduction

Respiratory syncytial virus (RSV) infection is a significant health threat, particularly for infants and the elderly. RSV-related lower respiratory tract infections (LRTI) cause approximately 3 million hospitalizations and 120,000 deaths annually among children < 5 years of age (Baraldi et al., 2022; Sadoff et al., 2022). In severe cases, RSV lead to bronchiolitis or pneumonia and heighten the risk of persistent respiratory complications, asthma onset, and impaired lung function (Kieffer et al., 2022). Unfortunately, genetic varieties, immune enhancement effect and the compromised immunity of the susceptible population limited the RSV vaccination (McLaughlin et al., 2022). Therefore, curative strategies against RSV are crucial to mitigate the potential harm of RSV infection.

Traditional Chinese medicine (TCM) has a history of use in the treatment of respiratory viral infections before (Li et al., 2021), often focusing on symptom relief, through performing immunomodulatory and anti-inflammatory roles that work in managing symptoms and supporting recovery. Herbs like Astragalus (Huangqi), Honeysuckle Flower (Jinyinhua), and Forsythia Fruit (Lianqiao) are commonly used in TCM formulas to clear heat, relieve sore throat, and alleviate fever (Tian and Huang, 2017; Ni et al., 2020; Xiong et al., 2020). The medicinal extracts of Ephedrae Herba and Cinnamomi Cortex specifically interact with the central conserved domain (CCD) of RSV G protein, which disrupts the attachment to the CX3CR1 receptor in host cell (Fujikane et al., 2022). Yin Qiao San, a classic TCM formula, is often employed for early-stage respiratory viral infections with symptoms like fever and sore throat. However, whether TCM has direct anti-RSV effects remains unknown.

Respiratory syncytial virus (RSV) and many other enveloped viruses depend on lipid rafts to realize their entry into host cells and virus particles assembly (Carrasco et al., 2004; Brown et al., 2005; Li et al., 2017). The viral envelope proteins interact with specific lipids and proteins in these rafts, facilitating the attachment and fusion of the viral particle with the host cell membrane (Chang et al., 2012). RSV exploits caveolae for its assembly in the lipid raft (Ludwig et al., 2017). Moreover, host cells cholesterol production was induced during RSV infection as the structural lipid components of lipid rafts (Bajimaya et al., 2017; Bakillah et al., 2022). As the most critical rate-limiting enzyme of cholesterol synthesis, HMGCR’s elevated activity during respiratory syncytial virus infection facilitates the actin-dependent transmission of the virus between cells (Ravi et al., 2013). SREBP2 controls the cholesterol homeostasis through a negative feedback loop, which the elevated cellular cholesterol levels by SREBP2 in turn inhibit SREBP2 cleavage for maturation leading to suppression of the expression of genes involving cholesterol synthesis and uptake (Guo et al., 2018). However, the exact mechanisms by which RSV regulates the activation of SREBP2 remain unknown. Some studies suggest that the enhanced expression of HMGB1 protein RSV may be responsible for SREBF2 activation in RSV infection (Najima et al., 2005; Manti et al., 2018; Ghaffari et al., 2020). Nevertheless, targeting cholesterol synthesis remains unvalidated as an effective anti-RSV strategy.

In this study, we found XFF inhibits RSV replication and its triggered inflammation response *via* suppressing cholesterol synthesis. XFF treatment reduced both serum pro-inflammation cytokines and RSV titers in RSV-inoculated mice. Further, Q-PCR and Immunohistochemistry (IHC) staining assays confirmed that XFF ameliorated the excessive activation of lung inflammation. Transcriptome analysis was adopted to explain the simultaneous reduced pro-inflammation response and RSV virus titers, and results pointed toward lipid metabolism. Lipidomics profiling showed consistent cholesterol abundance with transcriptional changes, other than fatty acids. The intermediates concentration and the expression of rate-limited enzyme of cholesterol synthesis also support XFF’s suppressive action on cholesterol synthesis. Lovastatin treatment *in vitro* mimicked this condition, inhibiting RSV replication and pro-inflammatory cytokine expression. Transcription factor prediction of differentially expressed genes implicated SREBP2 in cholesterol synthesis, while network pharmacology and molecular docking identified β-sitosterol and stigmasterol as promising active ingredients in XFF. *In vitro* experiments demonstrated that β-sitosterol and stigmasterol strengthened bonding between SCAP and INSIGs, inhibiting SREBP2 cleavage maturation and consequently impeding RSV infection. These findings underscore the potential of XFF in modulating host responses and lipid metabolism to mitigate RSV-associated pathology.

## Materials and methods

### Cells and viruses (RSV proliferation and cell culture)

Cells culture and RSV proliferation were performed as previously described (Luo et al., 2022). HEp-2 and Vero cell lines were purchased from the China Center for Type Culture Collection (CCTCC) in Wuhan, China, were cultured in Dulbecco’s modified Eagle’s medium (DMEM). The culture medium was supplemented with 10% fetal bovine serum (FBS, Gibco), and 1% Penicillin-Streptomycin liquid. The cells were incubated at a temperature of 37°C in an atmosphere containing 5% CO_2_. The respiratory syncytial virus (RSV) A2 strain, maintained in our laboratory, was used for experiments. The RSV-infected HEp-2 cells were subjected to sonication and then clarified through centrifugation at 1,200 *g* for 30 min at 4°C, and then concentrated via ultracentrifugation at 120,000 *g* for 6 h at the same temperature. The resulting supernatant was concentrated by ultracentrifugation at 120,000 *g* for 6 h at 4°C. Subsequently, the obtained pellet was reconstituted in phosphate-buffered saline (PBS) to facilitate RSV purification. For the purification of RSV, the pellet was suspended in PBS containing 10% sucrose and layered onto a discontinuous sucrose gradient consisting of three layers, each with 2 mL volume: 60, 45, and 30% sucrose (in PBS). The sample was then centrifuged at a speed of up to 160,000 *g*. The visible virus band, which was located between the 30 and 45% sucrose layers, was carefully collected for further experimentation.

### Preparation and testing of experimental drugs

Xuanfei Formula, comprising eight individual commercialized formula granules manufactured by CR SANJIU in Shenzhen, China. The proportions of these single formula granules to their corresponding raw herbal components are detailed in Supplementary Table 1.

In accordance with the Chinese Pharmacopoeia’s technical guidelines for assessing the quality and stability of traditional Chinese medicine preparations, we conducted ultrahigh-performance liquid chromatography (UPLC) analysis on the active ingredients corresponding to the main constituents. In this investigation, 5 g samples were precisely weighed and placed into 150 mL conical flasks. Thereafter, 50 mL of water at 60°C was added before ultrasonic dissolution. The resultant solution was then decanted into a 250 mL volumetric flask, subjected to 40 mL ultrasonic extraction with 80% methanol, and subsequently, filtered through a microporous membrane to a final volume of 50 mL. Quality assessment was conducted using high performance liquid chromatography (HPLC) in accordance with the Chinese Pharmacopoeia guidelines. Analysis was performed on a Thermo U3000 HPLC system (USA) equipped with a water-resistant AQ C18 column (4.6 × 250 mm, 5 μm), utilizing a gradient elution of acetonitrile and 0.1% phosphoric acid water. The flow rate was maintained at 1.0 mL/min, with a column temperature of 35°C, and chromatographic monitoring at 230 nm. For the specific quantification of platycodon D, the evaporative light scattering detector (ELSD) method, as prescribed by the Chinese Pharmacopoeia, was utilized. The peak profiles of the formula, synthesized by the commercialized granules from ten batches, and the RSD values (less than 10%) indicated their quality and stability (Supplementary Figures 1A, B and Supplementary Table 2).

### Animal modeling of RSV and grouping

Female specific-pathogen-free (SPF) BALB/c mice, aged between six to eight weeks, were procured from the Hubei Experimental Animal Research Center in Wuhan, China. The mice were divided into seven groups: Group Mock received no treatment; the remaining groups were simultaneously exposed to RSV infection and treated with Xuanfei or saline, each categorized based on their particular treatment type and duration. All mice were housed under SPF conditions and had unrestricted access to food and water. To initiate the experiment, mice were intranasally infected with 1 × 10^6^ plaque-forming units (PFU) of RSV in a 50 μL volume, while the Mock group received an equivalent volume of PBS. Xuanfei Formula or saline treatment (at a dosage of 5 g/kg/day) was administered intragastrically daily, with the first treatment starting in 4 h post-RSV infection.

### Histological analysis immunohistochemistry

The entire lung samples from the mice were collected at specified time points and then preserved in 4% paraformaldehyde before being embedded in paraffin for subsequent sectioning. These tissue sections were affixed onto glass slides, and for microscopic examination of histopathological alterations, they underwent staining with hematoxylin and eosin (H&E).

In the immunohistochemistry assay, lung tissue paraffin slides were initially deparaffinized using xylene and then gradually rehydrated through an ethanol gradient. Antigen retrieval was carried out using a 10 mM sodium citrate buffer (pH = 6), followed by blocking with 3% hydrogen peroxide (H_2_O_2_) and permeabilization using 1% bovine serum albumin (BSA). Overnight incubation at 4°C with either F4/80 antibody (diluted at 1:200, Abcam) or CD68 antibody (diluted at 1:200, Affinity). Following this, a secondary antibody (diluted at 1:200, Aspen) was applied and allowed to incubate at 37°C for 30 min. Post-DAB staining, sections were counterstained with hematoxylin for 10 min, dehydrated, and sealed using neutral resin.

### Real-time reverse transcription quantitative PCR (RT-qPCR)

To determine the RSV titer in the lung, we employed RT-qPCR as reported (Yang et al., 2020). Total RNA was extracted from lung tissues using RNA Pure reagent (manufactured by Aidlab, Beijing, China). Subsequently, we carried out reverse transcription to convert the RNA into complementary DNA (cDNA) using a reverse transcription kit from Toyoba in Osaka, Japan, following the instructions provided by the manufacturer. The RSV N gene copies was quantified utilizing 2 × SYBR green master mix from Novoprotein in Shanghai, China, and the analysis was performed on a 7500 Real-Time PCR System manufactured by Applied Biosystems in the USA. Specific primers of RT-qPCR are listed in Supplementary Table 3.

### ELISA

To determine the levels of cytokines in the lung tissues, we employed enzyme-linked immunosorbent assay (ELISA) following established procedures (Lei et al., 2021). Lung samples were collected at specified time intervals. These samples were then subjected to homogenization and subsequent centrifugation. The quantitative assessment of cytokines including IL-6, IL-8, and IL-17A in the lung homogenates was carried out using commercially available ELISA kits sourced from BioLegend and Biotechnology.

### Virus replication and cytokines assay in HEp-2 cells

HEp-2 cell monolayers, cultured in 12-well plates, were subjected to two different conditions: one group was treated with 10 μM lovastatin, while the other group remained untreated (Gower and Graham, 2001). This treatment or lack thereof began 24 h prior to the infection with RSV at a multiplicity of infection (MOI) of 1. Two hours after the infection, the experimental setup transitioned to using a medium containing 2% serum. To monitor the virus’s replication, daily assessments were made over the course of five consecutive days following RSV infection. For this evaluation, the entire contents of each well were harvested, subjected to freeze-thaw cycles, and then processed to extract RNA. Subsequently, RT-qPCR was employed to detect viral replication. In addition to assessing viral replication, cellular samples were collected for cytokine detection using RT-qPCR. Detailed information on the primer sequences for RT-qPCR can be found in Supplementary Table 3.

### RNA-seq

Total RNA was extracted using Trizol Reagent. The quality and quantity of the total RNA were assessed by the Agilent 2100/4200 system. For the preparation of RNA samples for sequencing, we initiated the process with sequencing libraries construction followed by mRNA isolation, fragmentation of mRNA, first and second cDNA strands synthesis, end repair, purification and quantification accomplished using Qubit. Subsequently, the library of the target region for the duplex was denatured, cycled, and digested to produce a single-stranded circular DNA. This single-stranded circular DNA was then amplified through rolling circle amplification. Post-library construction, Qubit was again employed for quantification. Finally, the sequencing library was subjected to sequencing on the DNBSEQ-T7 platform using the PE150 model at Bioyi Biotechnology Co., Ltd. in Wuhan, China.

### Transcriptome data analysis

We employed several bioinformatics tools and statistical methods for our gene expression analysis. Our methodology is summarized as follows.

Gene Expression Quantification: We initially used StringTie version 2.1.5 to calculate Read Count values for each gene, which represents the original gene expression level. These values were then standardized using FPKM (Fragments Per Kilobase Million). Differential Gene Expression Analysis: To identify genes with significant expression differences, we utilized DESeq2 version 1.30.1. We filtered genes based on specific criteria, including a fold change of expression (|FoldChange| > 1.5) and statistical significance (padj ≤ 0.05).

K-means Analysis: K-means clustering in RNA-seq data analysis groups genes or samples based on their expression patterns. It aids in identifying distinct patterns and simplifying complex datasets. We performed k-means analysis with the help of the R language package factoextra and cluster.

Gene Ontology (GO) Enrichment Analysis: To understand the biological functions associated with differentially expressed genes, we mapped these genes to GO database terms. Using Cluster Profiler, version 3.18.1, we conducted GO enrichment analysis and calculated *p*-values based on the hypergeometric distribution method. Significant enrichment was determined when padj ≤ 0.05.

This comprehensive analysis helped us understand the differential gene expression patterns and the biological functions and pathways associated with these genes in our dataset.

### Lipidomic analysis

The detailed information was obtained from the previous report (Narváez-Rivas and Zhang, 2016). To prepare lung tissue samples for comprehensive lipidomic profiling through liquid-liquid MTBE extraction, follow these steps: an appropriate sample volume was placed it into a 2 mL EP tube, adding 750 μL of pre-cooled (−20°C) chloroform-methanol mixed solution (2:1), vortex for 30 s, and grind with steel balls in a tissue grinder at 50 Hz for 60 s, repeating this step twice. Keep the tube on ice for 40 min, then add 190 μL of H_2_O, vortex for 30 s, and return it to ice for an additional 10 min. Centrifuge at room temperature at 12,000 rpm for 5 min, carefully transfer the lower layer fluid (300 μL) to a new centrifuge tube, and add 500 μL of pre-cooled (−20°C) chloroform-methanol mixed solution (2:1), vortex again for 30 s. Transfer the lower layer fluid to the same centrifuge tube after centrifugation at 12,000 rpm for 5 min at room temperature. Concentrate the samples to dryness under vacuum, dissolve in 200 μL of isopropanol, and filter the resulting supernatant through a 0.22 μm membrane for LC-MS analysis using a Thermo Q Exactive HF mass spectrometer.

For untargeted lipidomic analysis, employ a Thermo Q Exactive HF mass spectrometer in both positive and negative ion modes using a HESI source. Optimize tune parameters and use specific lipid standards for calibration. Set sheath gas and sweep gas flow rates, as well as the auxiliary gas rate, accordingly. Maintain spray voltage, capillary temperature, and heater temperature at specified levels, and use an S-Lens RF level of 50. Operate the Orbitrap mass analyzer at defined resolving powers in full-scan and data-dependent MS2 modes with dynamic exclusion.

Finally, use LipidSearch software (v4) to obtain raw data for lipid annotation individually. Create a data matrix containing mass-to-charge ratio (m/z), retention time (rt), and peak response value (intensity). Align annotation results from all samples through LipidSearch software (v4.0), and perform peak alignment and filtering across single data annotation results to generate a comprehensive lipidomic dataset Subsequently, normalized lipid profiles were further processed by k-means algorithm.

### Western blot analysis

Western blot was performed according to the standard protocol. Tissue lysis was achieved by treating them with RIPA buffer containing a complete protease inhibitor. The resulting lysates were subjected to centrifugation at 12,000 rpm for 5 min at a temperature of 4°C. To determine protein concentrations in the supernatants, we employed a BCA protein assay kit sourced from Aspen, Canada, USA.

Following quantification, the protein samples were separated on 10% SDS-PAGE gels and subsequently transferred onto PVDF membranes. These membranes were then subjected to a blocking step using a 5% solution of non-fat dried milk, which lasted for one hour. This was followed by a series of sequential incubations with primary antibodies and their corresponding horseradish peroxidase (HRP)-conjugated secondary antibodies. As a reference for loading control, β-actin was utilized. Protein bands were visualized using enhanced chemiluminescence (ECL) reagents and subsequently analyzed utilizing an imaging system provided by QImaging in Surrey, Canada. All the antibodies used here are listed in Supplementary Table 4.

### Active ingredient profiling

Active ingredients of the complex prescription composed of Jiegeng (Platycodon), Xinyi (Magnolia Flower), Baizhi (Dahurian Angelica), Wumei (Mume Fruit), Fangfeng (Saposhnikovia), Gancao (Licorice), Sangbaipi (Mulberry Root Bark), and Huangqin (Scutellaria) were screened separately using the TCMSP database.^1^ Predicted ingredients of XFF were listed in Supplementary Table 5.

### Molecular docking

Based on guided by transcriptomics and lipidomics studies, we identified SREBP2 as a pivotal player in cholesterol metabolism, serving as the target for our drug investigation and focused on SCAP-INSIG complex, causing its determinant regulation upon SREBP2’s proteolytic cleavage and nuclear translocation. Consequently, we utilized the SCAP-INSIG complex structure as the docking model for screening active ingredient from XFF. Our methodology included downloading the receptor’s tertiary structure from the PDB database and the drugs’ structures from PubChem. We employed Autodock for the docking process and used pymol for visualizing the results. Our selection criteria for outputs were based on lower binding free energy and the presence of hydrogen bonds, indicative of higher binding affinity.

### The identification of active ingredients in XFF

Sample solution preparation: 25 g of Xuanfei Formula was placed into a 50 mL volumetric flask, accurately weighed, and mixed with 45 mL of methanol, for ultrasonic extraction over 30 min. After cooling to room temperature, the volume was adjusted to 50 mL with methanol, thoroughly shaken, and 1 mL of the supernatant was transferred to a sample vial. This was then evaporated to dryness under nitrogen gas, and reconstituted with 1 mL of silylation reagent (anhydrous pyridine: hexamethyldisilazane: trimethylchlorosilane = 9:3:1), sealed, and heated at 60°C for 30 min. Following cooling, the sample was ready for analysis.

Standard solution preparation: 10 mg of stigmasterol and β-sitosterol reference standards were weighed into a 10 mL volumetric flask. This was dissolved in n-hexane and diluted to a final concentration of 1 mg/mL. Further dilution was performed to obtain a 500 μg/mL solution. Prior to analysis, treat as described above. GC-MS identification conditions: Agilent 7890A-5975C gas chromatography-mass spectrometry system (Santa Clara, CA, USA) equipped with an Agilent 19091S-433 chromatographic column (30 m × 250 μm × 0.25 μm). The inlet temperature was set at 300°C, and a split mode injection (split ratio 10:1) was employed. Helium gas (carrier gas) was maintained at a constant flow rate of 1.2 mL/min. The injection volume was 1 μL. The temperature program ranged from 230 to 300°C. The interface temperature was set at 300°C, the ion source temperature at 230°C, and the MS quadrupole at 150°C. The analysis included full scan mode (50–550 m/z) and selected ion monitoring mode.

### Detection of SREBP2 cleavage regulated by stigmasterol and β-sitosterol *in vitro*

Respiratory syncytial virus (RSV)-infected Hep-2 cell model was employed here. β-sitosterol and stigmasterol were dissolved in the medium at varying concentrations, with 25-HC serving as the positive control. After 24 h of incubation, cells were infected with RSV for 2 h. Following an additional 24-h incubation in 2% serum-containing medium, cells were harvested. RNA extraction was performed for virus qRT-PCR analysis, while protein extraction was carried out for Western blot.

### Statistical analysis

Statistical analyses were conducted using GraphPad Prism 8.0 and SPSS 21.0 software. Two-tailed Student’s *t*-tests were employed for normally distributed data to compare differences between two groups, while one-way analysis of variance (ANOVA) was utilized for comparisons among multiple groups. *Post hoc* analyses included Bonferroni analysis for homogenous variance or Tamhane’s T2 analysis for heteroscedasticity. Non-parametric statistical analysis using the Mann-Whitney U test was applied for non-normally distributed data. Results are presented as mean ± standard deviation, and significance levels were categorized as *p ≤ 0.05 and **p ≤ 0.01.

## Results

### XFF inhibited RSV replication and excessive systematic inflammation in the lung of mice

**FIGURE 1:**
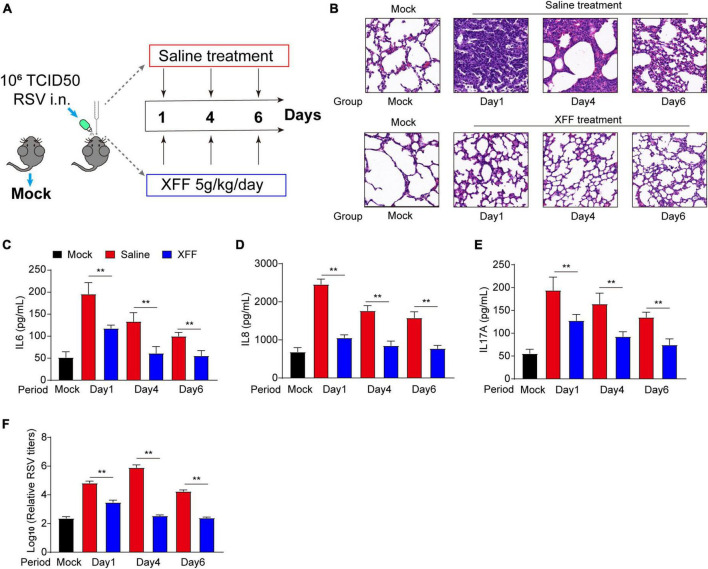
Inhibitory effects of XFF on RSV replication and excessive inflammation. **(A)** Schematic routine map of evaluation procedures for testing XFF’s anti-RSV effects in mice. **(B)** Histopathologic characteristics of lung sections from different groups by H&E staining. Scale bar indicates 200 μm. **(C)** Serum level of IL-6 among the indicated groups (*n* = 5, per group). **(D)** Serum level of IL-8 among the indicated groups (*n* = 5, per group). **(E)** Serum level of IL-17A among various groups (*n* = 5, per group). **(F)** RSV titers detected in the lysate of the lungs from various groups (*n* = 5, per group). Data are presented as mean ± SD and were analyzed using the student’s *t*-test. **Indicates *p* ≤ 0.01. XFF, Xuanfei Formula; RSV, respiratory syncytial virus; IL-6, interleukin-6; IL-8, interleukin-8; IL-17A, interleukin-17A.

The therapeutic efficacy of Xuanfei Formula (XFF) against respiratory syncytial virus (RSV) infection was assessed, as depicted in Figure 1A. Pathological images revealed pronounced thickening of alveolar and bronchial walls in the PBS group on the first day of infection, which gradually resolving over the subsequent week (Figure 1B). In contrast, the alveolar and bronchial wall remained normal in the mice treated with XFF throughout the entire validation period (Figure 1B). This pathological improvement correlated with changes in serum inflammatory cytokines. In detail, levels of pro-inflammatory factors, including interleukin (IL)-6, IL-8, and IL-17A, peaked on the first day of infection and gradually declined in the subsequent days (Figures 1C–F). Meanwhile, XFF treatment significantly attenuated the levels of these metrics compared to PBS treatment (Figures 1C–F). Notably, XFF administration markedly reduced RSV titers in pulmonary tissue homogenates. These results indicated XFF’s effect in mitigating RSV infection status.

The pro-inflammatory response serves as a double-edged sword during virus infection. An appropriate pro-inflammatory response acts as the trigger and indicator of adaptive immune response for virus clearance, while its hyperactivation can lead to tissue damage and sequelae (Habibi et al., 2020). We observed dual effectiveness of XFF in RSV titers and pro-inflammation cytokines, which evidently suggested XFF may have direct anti-RSV abilities. However, the underlying mechanism remains largely unknown.

### XFF suppresses the innate immune response in the early stages of RSV infection

**FIGURE 2:**
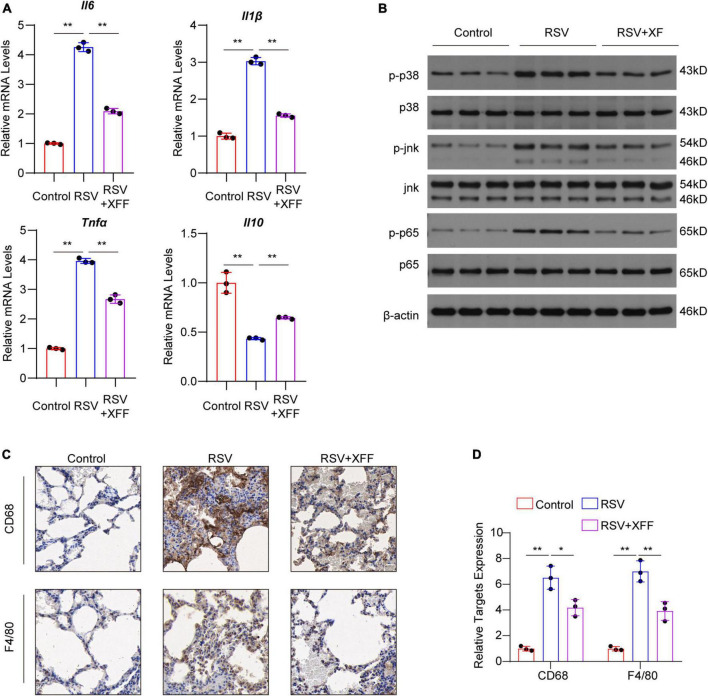
XFF alleviated early-stage lung inflammation induced by RSV infection. **(A)** Transcript level of pro-inflammation cytokines IL-1β, IL-6 and TNFα and anti-inflammatory cytokine IL-10 among control, model (RSV), and therapy groups (RSV+XFF) (*n* = 3, per group). **(B)** Western blot analysis of MAPK and NF-κB signaling pathway (*n* = 3, per group). **(C,D)** Represent IHC staining photographs **(C)** and its quantification statistics result **(D)** of the lung section from control, RSV, and RSV+XFF groups (*n* = 3, per group). Data are presented as mean ± SD and were analyzed using student’s *t*-test. **Indicates *p* ≤ 0.01. IL-10, interleukin-10; MAPK, mitogen-activated protein kinase; NF-κB, nuclear factor kappa-B.

On the initial day, we observed remarkable therapeutic efficacy against RSV replication, serum pro-inflammatory cytokines and alveolar wall thickening. Motivated by these findings, we checked pulmonary inflammatory responses status, a key contributor to severe RSV complications like pneumonia and asthma. In line with ELISA assays, the transcriptional levels of pro-inflammatory cytokines were significantly upregulated by RSV infection, and was mitigated by XFF treatment; while the anti-inflammatory cytokine IL-10 behaved the reverse trend (Figure 2A). Western blot analysis further indicated that XFF treatment reversed the excessive innate immune response such as MAPK and NF-κB signaling pathways initiated by RSV infection (Figure 2B). Excessive macrophage infiltration is a primary factor in adverse outcomes of RSV infection, such as pneumonia and asthma (Borchers et al., 2013). Immunohistochemistry staining for F4/80 (mature macrophage marker) and CD68 (macrophage marker) demonstrated RSV infection significantly increased the infiltration of macrophages in the lungs, whereas XFF treatment mitigated the pathological damage (Figures 2C, D). These findings underscored XFF’s anti-inflammatory effects during RSV infection. Nevertheless, the results of dampened innate immune response strongly suggests that the reduction in RSV titers may occur in an immune-independent manner once again. Further investigation is required to elucidate the mechanisms behind RSV replication suppression.

### Transcriptome analysis reveals XFF corrected lipid metabolism disruption caused by RSV infection

**FIGURE 3:**
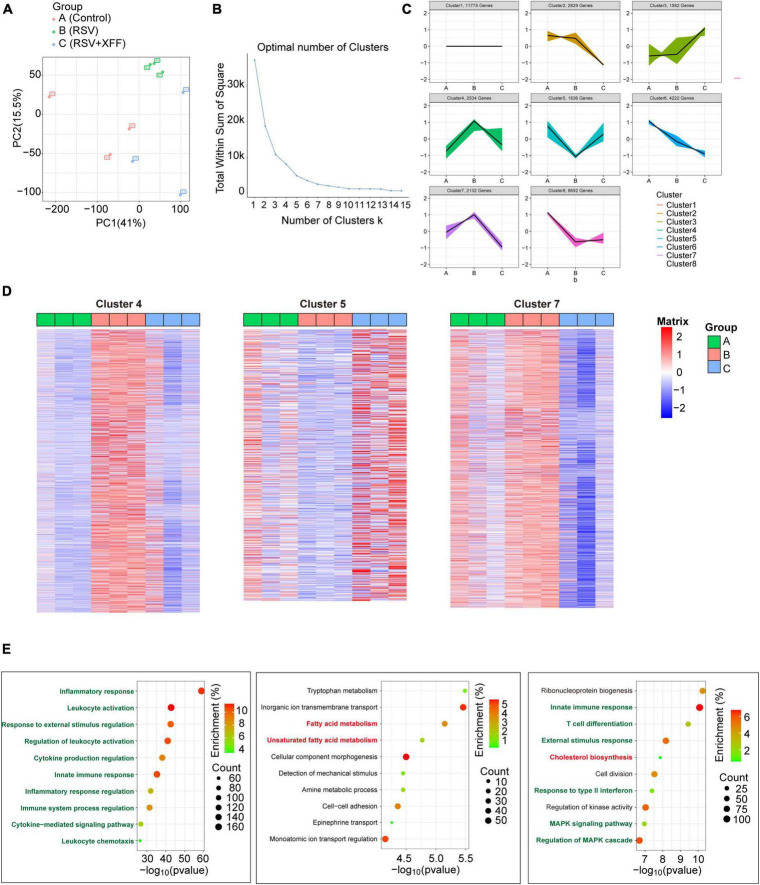
Transcriptome analysis in RSV-infected mice with XFF treatment. **(A)** Principal component analysis among control, model (RSV), and therapy groups (RSV+XFF) (*n* = 3, per group). **(B)** Elbow plot showing the sum of squared distances within clusters for different values of “k” (*n* = 3, per group). The optimal number of clusters is determined at the “elbow” point. **(C)** Visualization of the clustered data using a scatter plot illustrating the expression patterns of genes expression. Each color represents a distinct cluster identified by the K-means analysis (*n* = 3, per group). **(D)** Heatmap to illustrate the expression patterns of relevant genes across the interested clusters (*n* = 3, per group). **(E)** GO and WP enrichment plots of identified genes from the interested clusters (*n* = 3, per group). Data are presented as mean ± SD and were analyzed using student’s *t*-test. **Indicates *p* ≤ 0.01, *indicates 0.01 < *p* ≤ 0.05, and n.s. means *p* > 0.05.

To investigate the molecular mechanism of XFF suppressing RSV replication and improving pathological damage, we adopted transcriptome analysis. After data normalization, principal component analysis (PCA) was used to differentiate transcription profiles among the three groups (Figure 3A). With the guidance of elbow plot, we determined the optimal number of clusters as eight (Figure 3B). We subdivided the transcriptome data into eight clusters by the K-means analysis (Figure 3C). We specifically focused on those gene clusters in which RSV infection-brought expression changes were reversed by XFF treatment, as these genes were mostly likely to elucidate molecular mechanisms underlying XFF’s alleviation of RSV infection. To validate the reliability of the K-means analysis, the heatmaps were generated to examine the expression characteristics of each gene cluster (Figure 3D). Each cluster comprised approximately 1,000 genes, subjected to biological process enrichment analysis using Gene Ontology and Wikipedia algorithms. Among the biological process enrichment results, the predominantly altered processes were concentrated in innate immunity and subsequent adaptive immune response, aligning with our experimental results indicating that XFF treatment significantly reduces inflammation (Figure 3E). Notably, the cholesterol biosynthesis and fatty acid metabolism pathways appeared in clusters 7 and 5, respectively.

Disrupted lipid metabolism was indicated as the critical host responses to RSV and various lipid metabolic pathways were linked to disease pathology during RSV infection (Shan et al., 2018). Previous research established that hyperactive cholesterol synthesis is indispensable for cell fusion and assembly during RSV invasion and replication (Veit and Thaa, 2011; Bajimaya et al., 2017), while short-chain fatty acid acetate triggers antiviral response mediated by RIG-I and reduces RSV load consequently (Antunes et al., 2022). Therefore, we hypothesized that lipid metabolism alternation may be the reason for suppressed RSV replication following XFF treatment.

### Lipidomics analysis confirmed XFF reverses elevated cholesterol levels induced by RSV infection

**FIGURE 4:**
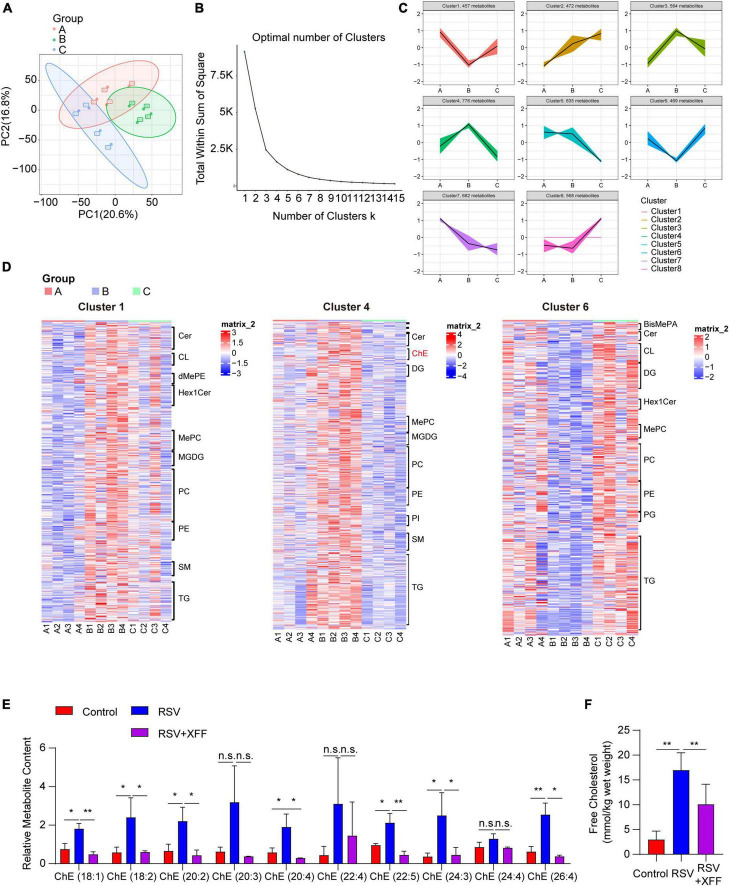
Lipidomic analysis of XFF against RSV-induced lipid alternation. **(A)** Principal component analysis among control, model (RSV), and therapy groups (RSV+XFF) (*n* = 5, per group). **(B)** Elbow plot showing the sum of squared distances within clusters for different values of “k” (*n* = 5, per group). The optimal number of clusters is determined at the “elbow” point. **(C)** Visualization of the clustered data using a scatter plot illustrating the abundance patterns of various lipids. Each color represents a distinct cluster identified by the K-means analysis (*n* = 5, per group). **(D)** Heatmap to illustrate the abundance patterns of relevant lipids across the interested clusters (*n* = 5, per group) and the top10 lipid constituents were named. **(E)** The concentration of cholesterol esters in lung tissues of mice among control, model (RSV), and therapy groups (RSV+XFF) (*n* = 5, per group). **(F)** The concentration of free cholesterol measurement in the RSV-infected murine lungs (*n* = 5, per group). Data are presented as mean ± SD and were analyzed using student’s *t*-test. **Indicates *p* ≤ 0.01, *indicates 0.01 < *p* ≤ 0.05, and n.s. means *p* > 0.05.

To substantiate role of lipid metabolism modulation for inhibiting RSV replication, we analyzed lipidomics profiling of lung tissues. Similarly, we initially performed PCA to discern lipid profiles among the three groups post- data normalization (Figure 4A). Guided by the elbow plot, we determined the optimal number of clusters as eight (Figure 4B) and subsequently employed K-means analysis to subdivide the lipidomics data into these eight clusters (Figure 4C). We specifically focus on lipid clusters where RSV-induced abundance changes were reversed by XFF treatment, as these lipids were likely to elucidate the molecular mechanisms underlying of XFF inhibited RSV infection. To validate the reliability of the K-means analysis, we employed expression heatmaps to examine the expression characteristics of each lipid cluster and meticulously listed the names of the top 10 lipid constituents (Figure 4D). The analysis revealed that only variations in cholesterol levels paralleled transcriptional changes, while the concentrations of fatty acid-derived triglyceride (TG) and diglyceride (DG) exhibited both upward and downward trends simultaneously (Figure 4D). Notably, we observed a substantial enrichment of cholesterol esters in cluster 6, which exhibited a similar trend to the transcriptional activities related to cholesterol synthesis. The dominant detected cholesterol esters increased in the PBS group but decreased after XFF administration. The bar chart depicting the abundance of major cholesterol lipid components further substantiates that XFF treatment alleviates the increased cholesterol levels induced by RSV infection (Figure 4E). Similarly, free cholesterol in mouse lungs showed a similar tendency (Figure 4F). The increased cholesterol synthesis during RSV infection has been widely reported, and the cholesterol-enriched lipid raft structures play a crucial role in the invasion and subsequent replication processes of RSV (Fleming et al., 2006). Therefore, we logically hypothesized that the immune-independent antiviral mechanism of XFF treatment may be dependent on cholesterol synthesis activity.

### XFF suppresses cholesterol synthesis upregulated by RSV infection

**FIGURE 5:**
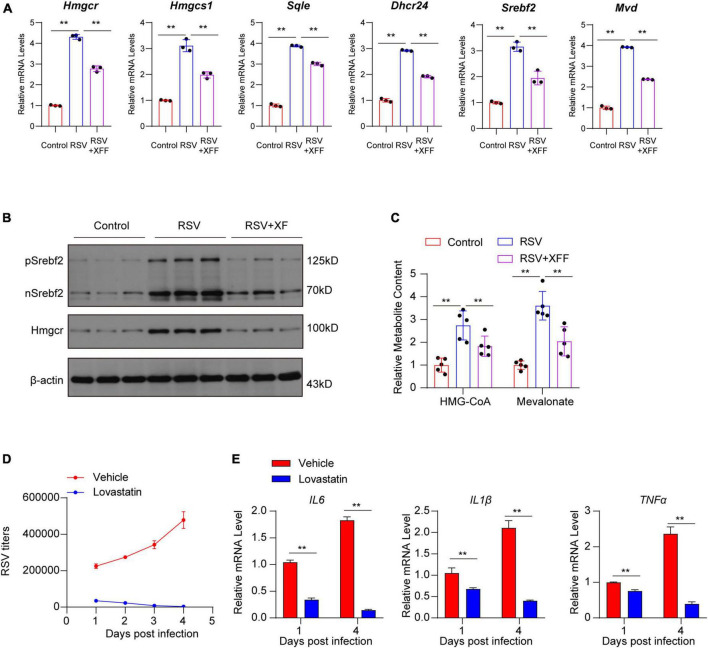
Dampened cholesterol synthesis suppressed RSV replication. **(A)** Relative mRNA levels of genes involved in cholesterol biosynthesis pathway in lungs of mice (*n* = 3 for each group). **(B)** Cleaved SREBP2 and HMGCR protein levels in lungs of mice (*n* = 3 for each group). **(C)** Concentration of cholesterol synthesis intermediates in lungs of mice (*n* = 3 for each group). **(D)** RSV titers in RSV-infected Hep-2 cell treated by vehicle or 5 μM lovastatin by RT-qPCR (*n* = 3 for each group). **(E)** Relative mRNA levels of pro-inflammation cytokines in RSV-infected Hep-2 cells treated by vehicle or 5 μM lovastatin (*n* = 3 for each group). Data are presented as mean ± SD and were analyzed using student’s *t*-test. **Indicates *p* ≤ 0.01.

To confirm XFF’s ability to inhibit RSV replication by suppressing cholesterol synthesis, we initially utilized qPCR to validate changes in the transcription levels of pivotal genes involved in cholesterol synthesis within the tissue. The results proved that XFF treatment effectively reversed the RSV-induced upregulation of genes related to cholesterol synthesis at both transcription (Figure 5A) and translation (Figure 5B) levels. Increased concentration of cholesterol synthesis intermediates induced by RSV infection was repressed by XFF administration (Figure 5C). To explore whether XFF-inhibited cholesterol synthesis results to RSV load decreasing, we assessed RSV replication and the secretion of pro-inflammatory cytokines in RSV-infected Hep-2 cells treated followed by cholesterol synthesis inhibitor lovastatin treatment *in vitro*. The results unequivocally indicated a significant inhibition of RSV replication within Hep-2 cells in the Lovastatin-treated group (Figure 5D). Likely, there was a notable reduction in the expression levels of pro-inflammatory cytokines (Figure 5E). Due to lacking RSV clearance system including macrophage, neutrophils or lymphocyte, the *in vitro* assay results intensely suggested the XFF-suppressed cholesterol synthesis can directly dampen RSV replication independent of immune system. In light of these findings, we substantiate that XFF suppresses RSV replication by inhibiting cholesterol synthesis, thereby ameliorating the effects of RSV infection.

### The phytosterol ingredients of XFF reduce cholesterol synthesis in SREBP2-dependent manner

**FIGURE 6:**
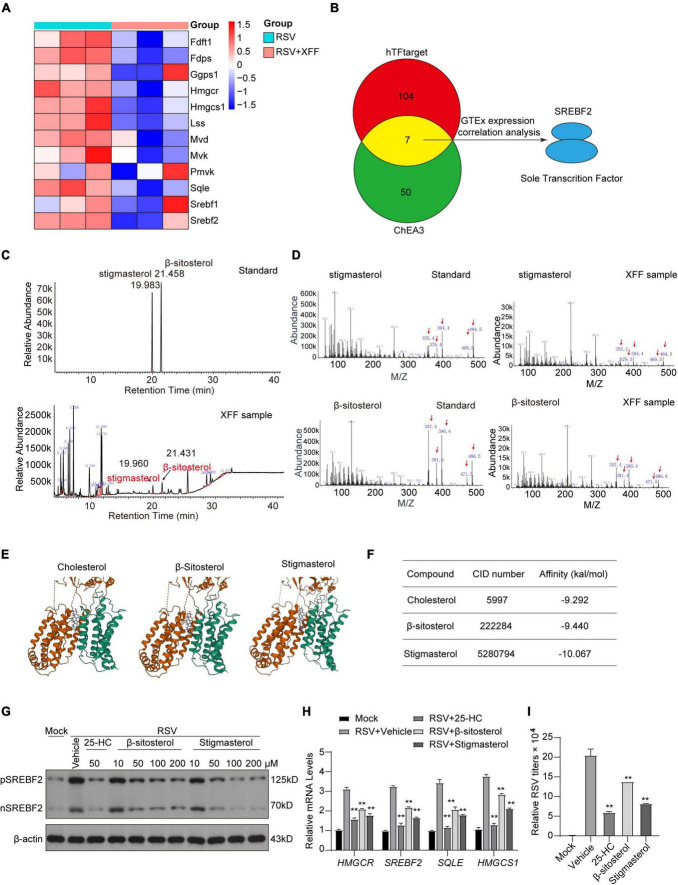
β-sitosterol and stigmasterol competitively bind to SCAP-INSIGs to suppress SREBP2-determinant cholesterol synthesis and RSV replication. **(A)** Heat map of genes involved in cholesterol synthesis in RSV-infected mice with or without XFF treatment. **(B)** Transcription factor prediction may co-regulate DEGs in **(A)** based on hTFtarget and ChEA3 database. **(C)** Gas chromatography map of β-sitosterol and stigmasterol standard in XFF extract. **(D)** Fragment ions of β-sitosterol and stigmasterol standard and the accordingly separated components in XFF extract. **(E)** The 3D digital model of molecular docking between cholesterol, β-sitosterol or stigmasterol and INSIGs-SCAP complex. **(F)** Table listed the affinity characteristics of cholesterol, β-sitosterol or stigmasterol with INSIGs-SCAP complex. **(G)** Cleaved SREBP2 and HMGCR protein levels in Hep-2 cells by Western blot analysis. **(H)** Relative transcription quantification of genes involved with cholesterol synthesis (** for the statistics significance between the group and RSV-Vehicle group). **(I)** RSV titers in RSV-infected Hep-2 cells treated with 25-HC, β-sitosterol or stigmasterol (** for the statistics significancy between the group and RSV-Vehicle group). Data are presented as mean ± SD and were analyzed using student’s *t*-test. **Indicates *p* ≤ 0.01.

We further investigated the specific active ingredient responsible for inhibiting cholesterol synthesis. Initially, we compared the DEGs in the cholesterol synthesis pathway in the lungs of RSV-infected mice with and without XFF treatment. Remarkably, XFF treatment was found to suppress the expression of 11 critical enzymes and 2 regulatory transcription factors in this pathway (Figure 6A). Subsequently, we conducted predictive analysis for the common transcription factors for the DEGs based on ChEA3 and HTFtargets databases and SREBP2 displayed higher correlation with transcriptional level of cholesterol synthesis in the candidate transcription factors (Figure 6B). Among these candidates, SREBP2 exhibited the strongest correlation with the expression levels of these differential genes. The SREBP2 activity is tightly controlled by the cholesterol content in ER: SCAP-SREBP2 complex translocate to Golgi for cleavage maturation under low cholesterol concentration (Supplementary Figure 2) and SREBP2’s function is likely affects by the phytosterol. Implied by the network pharmacology results, we employed gas chromatography-mass spectrometry (GC-MS) to identify 2 phytosterols in XFF, including β-sitosterol and stigmasterol. Gas chromatography first separated the components, with stigmasterol and β-sitosterol showing retention times of 19.960 and 21.423 min, respectively (Figure 6C). Following mass spectrometry detected the accurate identification based on characteristic mass fragments. The mass spectra display characteristic fragment ions for stigmasterol at M/Z 484, 469, 394, 379, and 355, and for β-sitosterol at M/Z 486, 471, 396, 381, and 357 as the red arrows labeled (Figure 6D). Based on the described retention times and characteristic fragment ions in the standard substance chromatograms, it is confirmed that XFF extract contains stigmasterol and sitosterol (Figures 6C, D). The data for the standard separated compounds have been verified according to the NIST2020 library. And molecular docking assay indicated β-sitosterol and stigmasterol dock strongly with INSIG-SCAP complex (Figure 6E), even stronger than cholesterol (Figure 6F). Both β-sitosterol and stigmasterol suppressed SREBP2 expression and cleavage mature in HEp-2 cells in dose-dependent way (Figure 6G). Treatment with 50 μM 2,5-hydroxycholesterol (25-HC), β-sitosterol, and stigmasterol repressed RSV-induced upregulation of genes involved in cholesterol biosynthesis in host cells (Figure 6H). Furthermore, a significant reduction in RSV titers was observed after treatment with β-sitosterol and stigmasterol, comparable to the effect of 25-HC (Figure 6I).

Collectively, our study identified key transcription factors and active ingredient within the compound responsible for inhibiting cholesterol synthesis, with SREBP2 emerging as a central player in this process. These findings shed light on the mechanisms underlying XFF’s impact on cholesterol synthesis and its therapeutic potential.

## Discussion

Globally, respiratory syncytial virus (RSV) predominantly affects infants, elderly individuals, and those with compromised immune systems (Shang et al., 2021). The COVID-19 pandemic’s non-pharmaceutical interventions initially disrupted the spread of most respiratory viruses, including RSV (Stein and Zar, 2023). Post-lifting of these restrictions, there was a notable rise in infant hospitalizations due to respiratory infections, often caused by RSV. Presently, no RSV vaccines are available for infants (Li Z. et al., 2022). The sole authorized preventive approach is the systemic use of anti-F (fusion) protein monoclonal antibodies, like palivizumab and motavizumab, which prevent RSV in premature babies but are not treatments for active infections (Sun M. et al., 2023).

Traditional Chinese medicine (TCM) is distinguished by a plethora of active ingredients and a multiplicity of targets. The formulation of XFF encompasses 207 active constituents, of which 185 are associated with efficacious pharmacological targets, cumulatively accounting for 270 distinct drug targets. Previous reports suggested XFF may mitigate RSV infection based on the demonstrated effectiveness of several active ingredients. For instance, quercetin exhibits anti-RSV properties due to the altered purine metabolism pathways (Sun Y. et al., 2023). Baicalin effectively obstructed RSV infection by inhibiting both cell attachment and intracellular replication processes during RSV infection (Shi et al., 2016). This study explored XFF’s effects on RSV-infected BALB/c mice, finding that early intervention with XFF significantly reduced pro-inflammatory cytokines and RSV replication, indicating an immune-independent anti-RSV effect. Transcriptome and lipidomic analysis revealed that XFF consistently normalized the hyperactivated cholesterol biosynthesis during RSV infection. Lovastatin treatment mimicked the protective effects of XFF against RSV *in vitro* without immune cells. Network pharmacology, molecular docking and mass spectrum analysis demonstrated that both β-sitosterol and stigmasterol in XFF are promising targets. Further experiments demonstrated that β-sitosterol and stigmasterol inhibited SREBP2 cleavage, finally hindering RSV infection. Our results hold clinical significance by uncovering the potential mechanisms of XFF in combating RSV infection, particularly its impact on cholesterol synthesis and consequent immune responses modulating.

In terms of host invasion and virus reproduction, there is accumulating evidence that lipid metabolism reprogramming of host cells plays critical roles (Antunes et al., 2020). Especially cholesterol metabolism, compelling evidence indicates that during various viral infections, including RSV and influenza, there is a notable induction of host cell cholesterol synthesis and plasma membrane cholesterol content change (Bajimaya et al., 2017; Ludwig et al., 2017; Lee et al., 2020). The increase in cholesterol synthesis in host cells has been proven to be essential for the infection process of various viruses, including RSV (Gower and Graham, 2001; Gutiérrez-Ortega et al., 2008). However, the regulatory mechanisms behind such a crucial phenomenon are largely unknown. In our study, we demonstrate that XFF can normalized the hyperactivated cleavage maturation of SREBF2 induced by RSV, which helps to exert antiviral activity against RSV that is independent of the immune system.

Mechanistically, the cholesterol-enriched lipid raft microdomain provides the binding, fusion and entry sites for virus infection (Yeo et al., 2009; Lee et al., 2020) and anchor sites for virus assembly (McDonald et al., 2004; Jumat et al., 2014). On the other hand, cholesterol also functions as signal molecules to stabilize or sensitize targets, which benefits the virus entry and replication (Ye, 2007; Palor et al., 2020). However, our research has only confirmed that the increased cholesterol synthesis is essential for RSV infection; there is a lack of study on the detailed molecular mechanisms, calling for further investigation.

While we propose that XFF exhibits antiviral activity independently of the immune system, the underlying mechanisms of its antiviral properties via direct viral interactions remain unaddressed. Indeed, the direct antiviral effects of synthetic and natural compounds are attracting increasing attention from drug development researchers. For instance, hydroxyketone derivatives (PF-00835231) with potent inhibitory effects on 3CLpro can suppress COVID-19 replication (Focosi et al., 2023). Azvudine acts as a dual-target inhibitor against HIV reverse transcriptase and the Vif protein, directly inhibiting HIV reverse transcription and assembly (Li G. et al., 2022). Furthermore, high-throughput screening results reveals that natural compounds hydroxyethyl phenol, hydroxybenzaldehyde, methyl dihydroxybenzoate decreased the activity of PLpro enzyme to 50–70%, in COVID-19 (Srinivasan et al., 2022). Therefore, subsequent research into the direct antiviral properties of XFF’s active gradients is imperative for developing more effective antiviral treatment strategies.

## Data availability statement

The transcriptome profiles can be found in the Gene Expression Omnibus with accession number GSE263754.

## Ethics statement

The animal study was approved by the Animal Care and Use Committee of the College of Life Sciences, Wuhan University. The study was conducted in accordance with the local legislation and institutional requirements.

## Author contributions

HQ: Data curation, Formal analysis, Investigation, Software, Writing – original draft, Writing – review & editing. JL: Investigation, Software, Writing – original draft. NZ: Conceptualization, Data curation, Investigation, Writing – original draft. WL: Investigation, Writing – original draft. PC: Validation, Software, Writing – original draft. HW: Formal analysis, Writing – original draft. ZP: Supervision, Writing – review & editing. XX: Funding acquisition, Supervision, Writing – review & editing.

## Footnotes

1
https://old.tcmsp-e.com/index.php
