# Supplementary material for: Xuanfei Formula inhibited RSV infection by normalizing the SREBP2-mediated cholesterol synthesis process
Source: Front Microbiol. 2024 May 2;15:1387062. doi: 10.3389/fmicb.2024.1387062 (PMC11100329; doi:10.3389/fmicb.2024.1387062)
Supplement: Supplementary file 1 [file Data_Sheet_1.docx]

**Supplementary Information**

**Supplementary Figures**


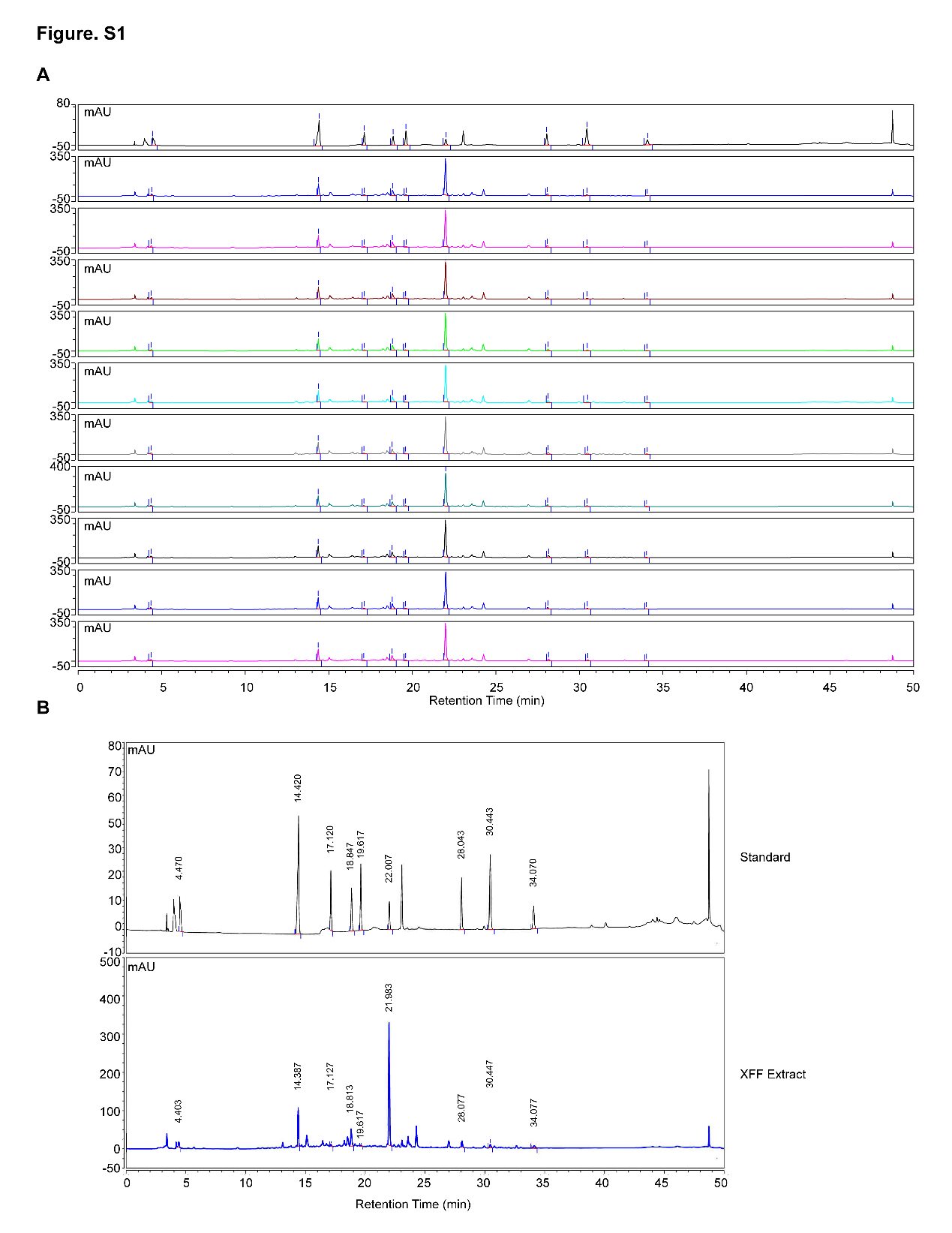


**Figure S1 Identification of anchored components of specific herbs from XFF**

**(A-B)** Liquid chromatography detection of anchored components of specific herbs from XFF, synthesized by granules across 10 batches (A) or from standard references (B).


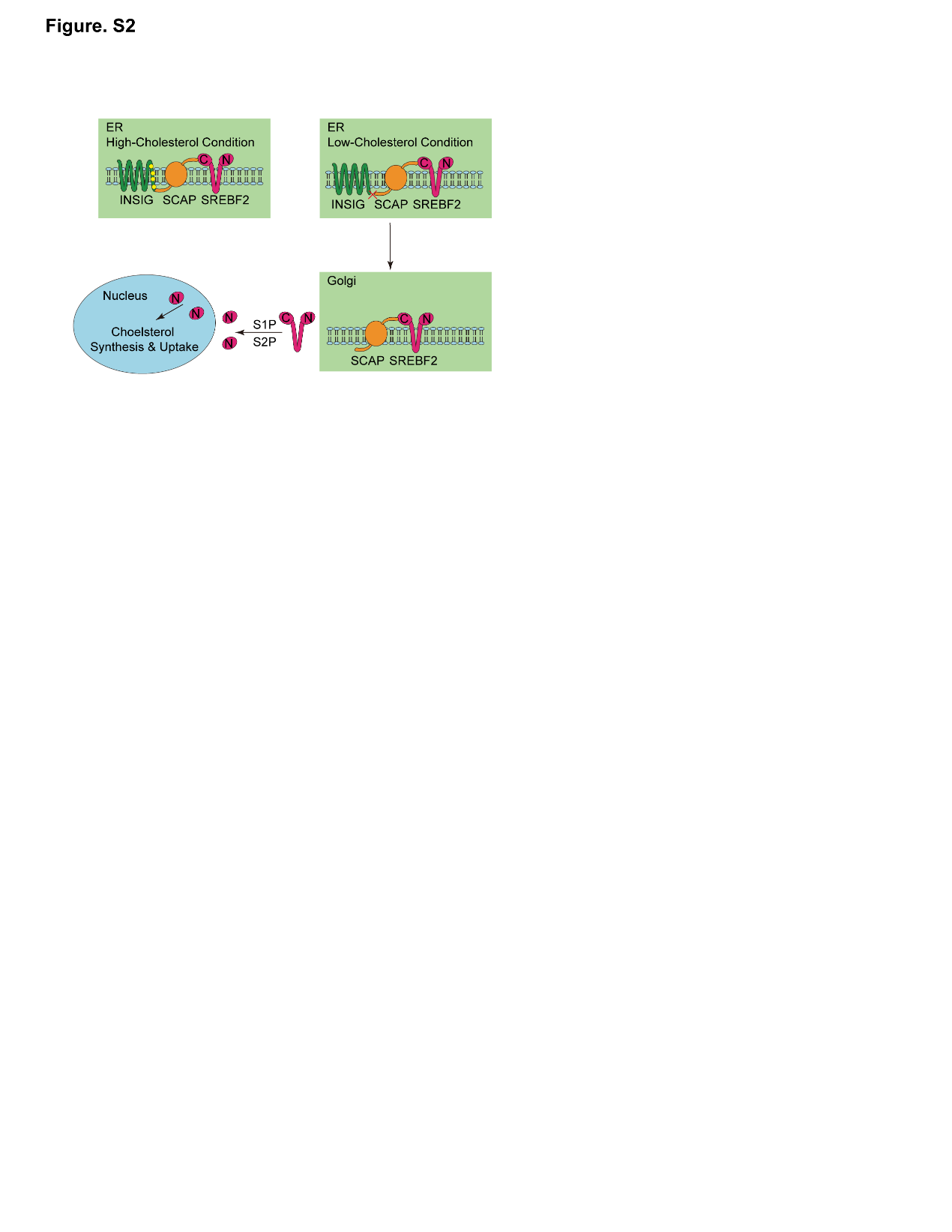


**Figure S2 Diagram of SREBF2-mediated cholesterol synthesis negative feedback regulation process**

**Table1 Composition of XFF**

| Granule name | English name | Amount (g) | Batch number |
| --- | --- | --- | --- |
| Radix Platycodonis | Balloonflower Root | 9 | 2102002S/2102003C/2102003S/2102004C/2102005C/2102006C/2102007C/2103002C/2107001S/2105001C |
| Radix Scutellariae | Baikal Skullcap Root | 9 | 2008007C/2008008C/2009003C/2009004C/2009005C/2009006C/2009007C/2011007C/2011008C/2011009C |
| Flos Magnoliae | Lily Magnolia | 9 | 2012003S/2102001C/2102002C/2102003C/2104001S/2104002S/2106001S/2106002S/2107002S/2107001S |
| Radix Angelicae Dahuricae | The Root of Dahurain Angelica | 9 | 2012002S/2012003S/2012004S/2102001S/2105001S/2105002S/2105003S/2105004S/2106001S/2106002S |
| Fructus Mume | Smoked Plum | 20 | 1910003C/1910001C/1910002C/1912002C/1912001C/2001002S/2001001S/2005001S/2005002S/2005003S |
| Radix Saposhnikoviae | Divaricate Saposhnikovia Root | 9 | 2002004C/2002005C/2002006C/2002007C/2002008C/2009002C/2009003C/2009004C/2012001C/2012002C |
| Radix Et Rhizoma Glycyrrhizae | Liquorice | 6 | 2004018C/2004019C/2004020C/2005001C/2005002C/2005003C/2005007C/2005008C/2005009C 2005010C |
| Cortex Mori | White Mulbrry Root-bark | 9 | 1911008C/2003001S/2003002S/2006001S/2006002S/2010001S/2010002S/2103001C/2103001S/2103002S |

**Table 2 Contents of anchored components of used granules in XFF from 10 batches**

| Granule name | Anchored components | Content in XFF across 10 batches(mg/g) | Average content (mg/g) | RSD (%) |
| --- | --- | --- | --- | --- |
| Radix Scutellariae | Baicalin | 33.67/29.87/32.17/34.07/32.55/30.18/35.72/31.94/34.66/36.67 | 33.15 | 6.73 |
| Flos Magnoliae | Magnolin | 1.44/1.28/1.31/1.45/1.29/1.39/1.43/1.55/1.27/1.49 | 1.39 | 7.04 |
| Radix Angelicae Dahuricae | Imperatorin | 0.17/0.16/0.16/0.18/0.16/0.17/0.18/0.15/0.17/0.18 | 0.168 | 6.14 |
| Fructus Mume | Citric Acid | 16.78/17.26/16.22/15.47/18.65/17.43/18.67/14.73/15.82/17.89 | 16.892 | 7.89 |
| Radix Saposhnikoviae | Prim-O-glucosylcimifugin | 0.75/0.74/0.78/0.73/0.77/0.82/0.67/0.83/0.69/0.77 | 0.755 | 6.73 |
| Saposhnikoviae | 5-O-Methylvisammioside | 0.95/1.02/1.06/0.89/0.96/1.13/0.99/0.98/1.14/1.01 | 1.013 | 7.76 |
| Radix Et Rhizoma Glycyrrhizae | Liquiritin | 3.56/3.22/3.68/3.46/3.68/3.41/3.76/3.53/3.49/3.27 | 3.506 | 5.01 |
| Glycyrrhizae | Ammonium Glycyrrhizinate | 3.21/3.56/3.48/3.69/3.14/3.53/3.27/3.49/3.36/3.66 | 3.439 | 5.45 |
| Cortex Mori | Mulberroside A | 7.59/7.68/7.52/7.43/8.34/8.24/7.99/8.42/8.17/7.88 | 7.926 | 4.53 |

**Table 3 Antibodies information**

| Name | Company | Lots |
| --- | --- | --- |
| anti-SREBF2 | Thermofisher | PA1-338 |
| HMGCR | Abcam | ab242315 |
| β-Actin | Tdybio | TDY051 |
| p-P38 | CST | #4511 |
| P38 | affbiotech | AF6456 |
| p-JNK | CST | #4668 |
| JNK | CST | #9252 |
| p-p65 | CST | #3033 |
| p65 | CST | #8242 |
| F4/80 | Abcam | ab300421 |
| CD68 | Servicebio | GB113109 |
| HRP-Goat anti Rabbit | ASPEN | AS1107 |
| HRP-Goat anti Mouse | ASPEN | AS1106 |

**Table 4 Primers sequences for qRT-PCR**

| Name | Sequence |
| --- | --- |
| *Hmgcr*-F | CTTGTGGAATGCCTTGTGATTG |
| *Hmgcr*-R | AGCCGAAGCAGCACATGAT |
| *Hmgcs1*-F | GCCGTGAACTGGGTCGAA |
| *Hmgcs1*-R | GCATATATAGCAATGTCTCCTGCAA |
| *Sqle*-F | CCTGTTGGGTTGCTTICAAT |
| Sqle-R | CACGTGGACTCCCTTTCAAT |
| *Dhcr24*-F | CATCTTCCGCTACCTCTTCG |
| *Dhcr24*-R | CTCTGCTTCATCTCCCTTGG |
| *Srebf2*-F | GCGTTCTGGAGACCATGGA |
| *Srebf2*-R | ACAAAGTTGCTCTGAAAACAAATCA |
| *Mvd*-F | AAGCAGACGGGCAGTACAGT |
| *Mvd*-R | CCTGGAGGTGTCATTGAGGT |
| *Il6*-F | AGTTGCCTTCTTGGGACTGA |
| *Il6*-R | TCCACGATTTCCCAGAGAAC |
| *Il1β*-F | CCGTGGACCTTCCAGGATGA |
| *Il1β*-R | GGGAACGTCACACACCAGCA |
| *Tnfα*-F | TGAATTCCCTGGGTGAGAAG |
| *Tnfα*-F | CTCTTCACCTGCTCCACTGC |
| *Il10*-F | CATCTTCTCAAAATTCGAGTGACAA |
| *Il10*-R | TGGGAGTAGACAAGGTACAACCC |
| *Actb*-F | GTGACGTTGACATCCGTAAAGA |
| *Actb*-R | GCCGGACTCATCGTACTCC |
| *IL6*-F | TCAGCCCTGAGAAAGGAGACAT |
| *IL6*-R | GCTCTGGCTTGTTCCTCACTACT |
| *IL1β*-F  *IL1β*-R  *TNFα*-F | ACGATGCACCTGTACGATCACT  GAGAACACCACTTGTTGCTCCA  CTCTTCTCCTTCCTGATCGTGG |
| *TNFα*-R | CTTGTCACTCGGGGTTCGAG |
| *HMGCR*-F | TACCATGTCAGGGGTACGTC |
| *HMGCR*-R | CAAGCCTAGAGACATAAT |
| *HMGCS1*-F | GATGTGGGAATTGTTGCCCTT |
| *HMGCS1*-R | ATTGTCTCTGTTCCAACTTCCAG |
| *SQLE*-F | TCCTTGCTCAGGCTCTTTATG |
| *SQLE*-R | AGGGTTAGGAGACAATACAGAAAG |
| RSV-N-F | AGATCAACTTCTGTCATCCAGCAA |
| RSV-N-R | TTCTGCACATCATAATTAGGAGTATCAAT |
| *ACTB*-F | GTCCACCGCAAATGCTTCTA |
| *ACTB*-R | TGCTGTCACCTTCACCGTTC |

**Table 5 Predicted ingredients of XFF**

| MOL_ID | Molecule_Name | OB | MW | Herb |
| --- | --- | --- | --- | --- |
| MOL001689 | acacetin | 34.97357273 | 284.28 | Balloonflower Root/Baikal Skullcap Root |
| MOL004580 | cis-Dihydroquercetin | 66.43699795 | 304.27 | Balloonflower Root |
| MOL005996 | 2-O-methyl-3-O-β-D-glucopyranosyl platycogenate A | 45.15023343 | 739.01 | Balloonflower Root |
| MOL000006 | luteolin | 36.16262934 | 286.25 | Balloonflower Root |
| MOL006026 | dimethyl 2-O-methyl-3-O-a-D-glucopyranosyl platycogenate A | 39.20757664 | 739.01 | Balloonflower Root |
| MOL006070 | robinin | 39.84373106 | 592.6 | Balloonflower Root |
| MOL012112 | 36150-23-9 | 34.73361611 | 370.43 | Lily Magnolia |
| MOL012119 | (1R,5R,6R,7R)-3-allyl-6-(3,4-dimethoxyphenyl)-1-methoxy-7-methylbicyclo[3.2.1]oct-2-ene-4,8-dione | 61.56303188 | 356.45 | Lily Magnolia |
| MOL012120 | (1R,5R,6R,7R)-3-allyl-6-(1,3-benzodioxol-5-yl)-1-methoxy-7-methylbicyclo[3.2.1]oct-2-ene-4,8-dione | 58.7854707 | 340.4 | Lily Magnolia |
| MOL012123 | denudanolide a | 31.19320504 | 354.43 | Lily Magnolia |
| MOL012124 | denudanolide b | 100.0598851 | 356.45 | Lily Magnolia |
| MOL012125 | denudanolide c | 62.5604696 | 370.48 | Lily Magnolia |
| MOL012126 | denudanolide d | 51.27551134 | 400.51 | Lily Magnolia |
| MOL012129 | 5-[(1R,3aR,4S,6aR)-4-(3,4-dimethoxyphenyl)-1,3,3a,4,6,6a-hexahydrofuro[4,3-c]furan-1-yl]-1,3-benzodioxole | 55.61475137 | 370.43 | Lily Magnolia |
| MOL012130 | (2S,3R,3aR,7R,7aS)-7-allyl-2-(1,3-benzodioxol-5-yl)-3a,4-dimethoxy-3-methyl-2,3,7,7a-tetrahydrobenzofuran-6-one | 59.3731376 | 372.45 | Lily Magnolia |
| MOL012131 | isodihydrofutoquinol a | 60.5438452 | 356.45 | Lily Magnolia |
| MOL012136 | 5-[(2S,3S,4R,5R)-5-(3,4-dimethoxyphenyl)-3,4-dimethyl-2-tetrahydrofuranyl]-1,3-benzodioxole | 60.1146053 | 356.45 | Lily Magnolia |
| MOL012137 | magnolone | 50.55684919 | 388.45 | Lily Magnolia |
| MOL000310 | Denudatin B | 61.47237606 | 356.45 | Lily Magnolia |
| MOL000313 | Galgravin | 57.11892168 | 372.5 | Lily Magnolia |
| MOL000314 | (2S,3S,4S,5S)-2,5-bis(3,4-dimethoxyphenyl)-3,4-dimethyltetrahydrofuran | 57.11892168 | 372.5 | Lily Magnolia |
| MOL000315 | hancinone | 39.31244 | 340.4 | Lily Magnolia |
| MOL007563 | Yangambin | 57.52544673 | 446.54 | Lily Magnolia |
| MOL009849 | ZINC05223929 | 31.5711208 | 354.38 | Lily Magnolia |
| MOL001494 | Mandenol | 41.99620045 | 308.56 | Lily Magnolia/The Root of Dahurain Angelica/Divaricate Saposhnikovia Root |
| MOL001939 | Alloisoimperatorin | 34.80406732 | 270.3 | The Root of Dahurain Angelica |
| MOL001941 | Ammidin | 34.54856394 | 270.3 | The Root of Dahurain Angelica/Divaricate Saposhnikovia Root |
| MOL001942 | isoimperatorin | 45.46424674 | 270.3 | The Root of Dahurain Angelica/Divaricate Saposhnikovia Root |
| MOL001956 | Cnidilin | 32.68737158 | 300.33 | The Root of Dahurain Angelica |
| MOL002883 | Ethyl oleate (NF) | 32.39738821 | 310.58 | The Root of Dahurain Angelica |
| MOL005789 | neobyakangelico l | 36.18178595 | 316.33 | The Root of Dahurain Angelica |
| MOL005792 | {5-[2'(R)-Hydroxy-3'-methyl-3'-butenyl-oxy]furocoumarin} | 42.85090514 | 286.3 | The Root of Dahurain Angelica |
| MOL005800 | Byakangelicol | 41.42285227 | 316.33 | The Root of Dahurain Angelica |
| MOL005802 | propyleneglycol monoleate | 37.602784 | 340.61 | The Root of Dahurain Angelica |
| MOL005806 | 4-[(2S)-2,3-dihydroxy-3-methylbutoxy]furo[3,2-g]chromen-7-one | 39.98526697 | 304.32 | The Root of Dahurain Angelica |
| MOL005807 | sen-byakangelicol | 58.00427462 | 386.43 | The Root of Dahurain Angelica |
| MOL000358 | beta-sitosterol | 36.91390583 | 414.79 | The Root of Dahurain Angelica/Smoked Plum/Divaricate Saposhnikovia Root/White Mulbrry Root-bark/Baikal Skullcap Root |
| MOL000449 | Stigmasterol | 43.82985158 | 412.77 | The Root of Dahurain Angelica/Smoked Plum/Baikal Skullcap Root |
| MOL000953 | CLR | 37.87389754 | 386.73 | The Root of Dahurain Angelica/Smoked Plum |
| MOL001506 | Supraene | 33.54594264 | 410.8 | The Root of Dahurain Angelica/Baikal Skullcap Root |
| MOL001749 | ZINC03860434 | 43.59332547 | 390.62 | The Root of Dahurain Angelica |
| MOL002644 | Phellopterin | 40.18555771 | 300.33 | The Root of Dahurain Angelica/Divaricate Saposhnikovia Root |
| MOL003588 | Prangenidin | 36.31449424 | 270.3 | The Root of Dahurain Angelica/Divaricate Saposhnikovia Root |
| MOL003791 | Linolein, 2-mono- | 37.28121005 | 354.59 | The Root of Dahurain Angelica |
| MOL007514 | methyl icosa-11,14-dienoate | 39.6670588 | 322.59 | The Root of Dahurain Angelica/Divaricate Saposhnikovia Root |
| MOL013430 | Prangenin | 43.59734075 | 286.3 | The Root of Dahurain Angelica |
| MOL001040 | (2R)-5,7-dihydroxy-2-(4-hydroxyphenyl)chroman-4-one | 42.36332114 | 272.27 | Smoked Plum |
| MOL000422 | kaempferol | 41.88224954 | 286.25 | Smoked Plum/Liquorice /White Mulbrry Root-bark |
| MOL005043 | campest-5-en-3beta-ol | 37.57681789 | 400.76 | Smoked Plum/White Mulbrry Root-bark |
| MOL008601 | Methyl arachidonate | 46.89969301 | 318.55 | Smoked Plum |
| MOL000098 | quercetin | 46.43334812 | 302.25 | Smoked Plum/Liquorice /White Mulbrry Root-bark/White Mulbrry Root-bark |
| MOL000011 | (2R,3R)-3-(4-hydroxy-3-methoxy-phenyl)-5-methoxy-2-methylol-2,3-dihydropyrano[5,6-h][1,4]benzodioxin-9-one | 68.82559903 | 386.38 | Divaricate Saposhnikovia Root |
| MOL011730 | 11-hydroxy-sec-o-beta-d-glucosylhamaudol_qt | 50.2435133 | 292.31 | Divaricate Saposhnikovia Root |
| MOL011732 | anomalin | 59.65405908 | 426.5 | Divaricate Saposhnikovia Root |
| MOL011737 | divaricatacid | 86.99614331 | 320.32 | Divaricate Saposhnikovia Root |
| MOL011740 | divaricatol | 31.65264449 | 334.35 | Divaricate Saposhnikovia Root |
| MOL011747 | ledebouriellol | 32.0501451 | 374.42 | Divaricate Saposhnikovia Root |
| MOL011749 | phelloptorin | 43.38603877 | 300.33 | Divaricate Saposhnikovia Root |
| MOL011753 | 5-O-Methylvisamminol | 37.9900618 | 290.34 | Divaricate Saposhnikovia Root |
| MOL000359 | sitosterol | 36.91390583 | 414.79 | Divaricate Saposhnikovia Root/Liquorice /Baikal Skullcap Root |
| MOL000173 | wogonin | 30.68456706 | 284.28 | Divaricate Saposhnikovia Root/Baikal Skullcap Root |
| MOL013077 | Decursin | 39.26720593 | 328.39 | Divaricate Saposhnikovia Root |
| MOL001484 | Inermine | 75.18306038 | 284.28 | Liquorice |
| MOL001792 | DFV | 32.76272375 | 256.27 | Liquorice |
| MOL000211 | Mairin | 55.37707338 | 456.78 | Liquorice /White Mulbrry Root-bark |
| MOL002311 | Glycyrol | 90.77578223 | 366.39 | Liquorice |
| MOL000239 | Jaranol | 50.82881677 | 314.31 | Liquorice |
| MOL002565 | Medicarpin | 49.21981761 | 270.3 | Liquorice |
| MOL000354 | isorhamnetin | 49.60437705 | 316.28 | Liquorice |
| MOL003656 | Lupiwighteone | 51.63569181 | 338.38 | Liquorice |
| MOL003896 | 7-Methoxy-2-methyl isoflavone | 42.56474148 | 266.31 | Liquorice |
| MOL000392 | formononetin | 69.67388061 | 268.28 | Liquorice |
| MOL000417 | Calycosin | 47.75182783 | 284.28 | Liquorice |
| MOL004328 | naringenin | 59.29389773 | 272.27 | Liquorice |
| MOL004805 | (2S)-2-[4-hydroxy-3-(3-methylbut-2-enyl)phenyl]-8,8-dimethyl-2,3-dihydropyrano[2,3-f]chromen-4-one | 31.78703353 | 390.51 | Liquorice |
| MOL004806 | euchrenone | 30.28726099 | 406.56 | Liquorice |
| MOL004808 | glyasperin B | 65.22438608 | 370.43 | Liquorice |
| MOL004810 | glyasperin F | 75.83680013 | 354.38 | Liquorice |
| MOL004811 | Glyasperin C | 45.56380662 | 356.45 | Liquorice |
| MOL004814 | Isotrifoliol | 31.94478724 | 298.26 | Liquorice |
| MOL004815 | (E)-1-(2,4-dihydroxyphenyl)-3-(2,2-dimethylchromen-6-yl)prop-2-en-1-one | 39.61685537 | 322.38 | Liquorice |
| MOL004820 | kanzonols W | 50.48007599 | 336.36 | Liquorice |
| MOL004824 | (2S)-6-(2,4-dihydroxyphenyl)-2-(2-hydroxypropan-2-yl)-4-methoxy-2,3-dihydrofuro[3,2-g]chromen-7-one | 60.25040908 | 384.41 | Liquorice |
| MOL004827 | Semilicoisoflavone B | 48.77755194 | 352.36 | Liquorice |
| MOL004828 | Glepidotin A | 44.72187465 | 338.38 | Liquorice |
| MOL004829 | Glepidotin B | 64.46292386 | 340.4 | Liquorice |
| MOL004833 | Phaseolinisoflavan | 32.00810772 | 324.4 | Liquorice |
| MOL004835 | Glypallichalcone | 61.59706227 | 284.33 | Liquorice |
| MOL004838 | 8-(6-hydroxy-2-benzofuranyl)-2,2-dimethyl-5-chromenol | 58.43728091 | 308.35 | Liquorice |
| MOL004841 | Licochalcone B | 76.75735485 | 286.3 | Liquorice |
| MOL004848 | licochalcone G | 49.25496332 | 354.43 | Liquorice |
| MOL004849 | 3-(2,4-dihydroxyphenyl)-8-(1,1-dimethylprop-2-enyl)-7-hydroxy-5-methoxy-coumarin | 59.62247498 | 368.41 | Liquorice |
| MOL004855 | Licoricone | 63.57845938 | 382.44 | Liquorice |
| MOL004856 | Gancaonin A | 51.07519107 | 352.41 | Liquorice |
| MOL004857 | Gancaonin B | 48.79440201 | 368.41 | Liquorice |
| MOL004860 | licorice glycoside E | 32.88743479 | 693.71 | Liquorice |
| MOL004863 | 3-(3,4-dihydroxyphenyl)-5,7-dihydroxy-8-(3-methylbut-2-enyl)chromone | 66.37125046 | 354.38 | Liquorice |
| MOL004864 | 5,7-dihydroxy-3-(4-methoxyphenyl)-8-(3-methylbut-2-enyl)chromone | 30.48877673 | 352.41 | Liquorice |
| MOL004866 | 2-(3,4-dihydroxyphenyl)-5,7-dihydroxy-6-(3-methylbut-2-enyl)chromone | 44.15196126 | 354.38 | Liquorice |
| MOL004879 | Glycyrin | 52.60657166 | 382.44 | Liquorice |
| MOL004882 | Licocoumarone | 33.21085068 | 340.4 | Liquorice |
| MOL004883 | Licoisoflavone | 41.61021885 | 354.38 | Liquorice |
| MOL004884 | Licoisoflavone B | 38.92870888 | 352.36 | Liquorice |
| MOL004885 | licoisoflavanone | 52.46624706 | 354.38 | Liquorice |
| MOL004891 | shinpterocarpin | 80.29527688 | 322.38 | Liquorice |
| MOL004898 | (E)-3-[3,4-dihydroxy-5-(3-methylbut-2-enyl)phenyl]-1-(2,4-dihydroxyphenyl)prop-2-en-1-one | 46.26792256 | 340.4 | Liquorice |
| MOL004903 | liquiritin | 65.69011165 | 418.43 | Liquorice |
| MOL004904 | licopyranocoumarin | 80.36001331 | 384.41 | Liquorice |
| MOL004905 | 3,22-Dihydroxy-11-oxo-delta(12)-oleanene-27-alpha-methoxycarbonyl-29-oic acid | 34.31942477 | 512.75 | Liquorice |
| MOL004907 | Glyzaglabrin | 61.06888631 | 298.26 | Liquorice |
| MOL004908 | Glabridin | 53.24514328 | 324.4 | Liquorice |
| MOL004910 | Glabranin | 52.89565508 | 324.4 | Liquorice |
| MOL004911 | Glabrene | 46.26685721 | 322.38 | Liquorice |
| MOL004912 | Glabrone | 52.51217419 | 336.36 | Liquorice /White Mulbrry Root-bark |
| MOL004913 | 1,3-dihydroxy-9-methoxy-6-benzofurano[3,2-c]chromenone | 48.14154235 | 298.26 | Liquorice |
| MOL004914 | 1,3-dihydroxy-8,9-dimethoxy-6-benzofurano[3,2-c]chromenone | 62.90135486 | 328.29 | Liquorice |
| MOL004915 | Eurycarpin A | 43.27728425 | 338.38 | Liquorice |
| MOL004917 | glycyroside | 37.25031932 | 562.57 | Liquorice |
| MOL004924 | (-)-Medicocarpin | 40.99397199 | 432.46 | Liquorice |
| MOL004935 | Sigmoidin-B | 34.88108616 | 356.4 | Liquorice |
| MOL004941 | (2R)-7-hydroxy-2-(4-hydroxyphenyl)chroman-4-one | 71.12298901 | 256.27 | Liquorice |
| MOL004945 | (2S)-7-hydroxy-2-(4-hydroxyphenyl)-8-(3-methylbut-2-enyl)chroman-4-one | 36.56537233 | 324.4 | Liquorice |
| MOL004948 | Isoglycyrol | 44.69922568 | 366.39 | Liquorice |
| MOL004949 | Isolicoflavonol | 45.16999058 | 354.38 | Liquorice |
| MOL004957 | HMO | 38.3654238 | 268.28 | Liquorice |
| MOL004959 | 1-Methoxyphaseollidin | 69.98097678 | 354.43 | Liquorice |
| MOL004961 | Quercetin der. | 46.4493884 | 330.31 | Liquorice |
| MOL004966 | 3'-Hydroxy-4'-O-Methylglabridin | 43.71495141 | 354.43 | Liquorice |
| MOL000497 | licochalcone a | 40.78965199 | 338.43 | Liquorice |
| MOL004974 | 3'-Methoxyglabridin | 46.16150929 | 354.43 | Liquorice |
| MOL004978 | 2-[(3R)-8,8-dimethyl-3,4-dihydro-2H-pyrano[6,5-f]chromen-3-yl]-5-methoxyphenol | 36.21429208 | 338.43 | Liquorice |
| MOL004980 | Inflacoumarin A | 39.70909598 | 322.38 | Liquorice |
| MOL004985 | icos-5-enoic acid | 30.70294255 | 310.58 | Liquorice |
| MOL004988 | Kanzonol F | 32.46833364 | 420.54 | Liquorice |
| MOL004989 | 6-prenylated eriodictyol | 39.22383018 | 356.4 | Liquorice |
| MOL004990 | 7,2',4'-trihydroxy－5-methoxy-3－arylcoumarin | 83.71436744 | 300.28 | Liquorice |
| MOL004991 | 7-Acetoxy-2-methylisoflavone | 38.92333105 | 294.32 | Liquorice |
| MOL004993 | 8-prenylated eriodictyol | 53.79476318 | 356.4 | Liquorice |
| MOL004996 | gadelaidic acid | 30.70294255 | 310.58 | Liquorice |
| MOL000500 | Vestitol | 74.65518912 | 272.32 | Liquorice |
| MOL005000 | Gancaonin G | 60.43520506 | 352.41 | Liquorice |
| MOL005001 | Gancaonin H | 50.10372327 | 420.49 | Liquorice |
| MOL005003 | Licoagrocarpin | 58.81390287 | 338.43 | Liquorice |
| MOL005007 | Glyasperins M | 72.67080984 | 368.41 | Liquorice |
| MOL005008 | Glycyrrhiza flavonol A | 41.27527733 | 370.38 | Liquorice |
| MOL005012 | Licoagroisoflavone | 57.28224098 | 336.36 | Liquorice |
| MOL005013 | 18α-hydroxyglycyrrhetic acid | 41.16138694 | 486.76 | Liquorice |
| MOL005016 | Odoratin | 49.94821817 | 314.31 | Liquorice |
| MOL005017 | Phaseol | 78.76621925 | 336.36 | Liquorice |
| MOL005018 | Xambioona | 54.84916242 | 388.49 | Liquorice |
| MOL005020 | dehydroglyasperins C | 53.82326014 | 340.4 | Liquorice |
| MOL012681 | Dimethyl (methylenedi-4,1-phenylene)biscarbamate | 50.84052189 | 314.37 | White Mulbrry Root-bark |
| MOL012686 | 7-methoxy-5,4'-dihydroxyflavanonol | 51.7201767 | 302.3 | White Mulbrry Root-bark |
| MOL012689 | cyclomulberrochromene | 36.78832377 | 418.47 | White Mulbrry Root-bark |
| MOL012692 | kuwanon D | 31.092096 | 422.51 | White Mulbrry Root-bark |
| MOL012714 | Moracin A | 64.38875088 | 286.3 | White Mulbrry Root-bark |
| MOL012717 | moracin M-6,3'-di-O-β-D-glucopyranoside | 37.80941867 | 566.56 | White Mulbrry Root-bark |
| MOL012719 | moracin O | 62.32610687 | 326.37 | White Mulbrry Root-bark |
| MOL012726 | mulberrofuran G | 92.19343575 | 562.6 | White Mulbrry Root-bark |
| MOL012735 | mulberroside C_qt | 71.3920742 | 326.37 | White Mulbrry Root-bark |
| MOL012743 | resveratrol-3,4'-di-O-β-D-glucopyranoside | 35.07599504 | 552.58 | White Mulbrry Root-bark |
| MOL012749 | sanggenone B | 115.4409787 | 570.63 | White Mulbrry Root-bark |
| MOL012753 | sanggenone F | 62.4237709 | 354.38 | White Mulbrry Root-bark |
| MOL012755 | sanggenone H | 37.50420812 | 354.38 | White Mulbrry Root-bark |
| MOL012760 | sanggenone M | 68.28888158 | 436.49 | White Mulbrry Root-bark |
| MOL001474 | sanguinarine | 37.80767752 | 332.35 | White Mulbrry Root-bark |
| MOL003758 | Iristectorigenin (9CI) | 71.54841214 | 330.31 | White Mulbrry Root-bark |
| MOL003856 | Moracin B | 55.85087738 | 286.3 | White Mulbrry Root-bark |
| MOL003857 | Moracin C | 82.13154641 | 310.37 | White Mulbrry Root-bark |
| MOL003858 | Moracin D | 60.92843177 | 308.35 | White Mulbrry Root-bark |
| MOL003860 | Moracin F | 53.81210736 | 286.3 | White Mulbrry Root-bark |
| MOL001004 | pelargonidin | 37.98831233 | 271.26 | White Mulbrry Root-bark |
| MOL012800 | 3,5,7-trihydroxy-2-(3-hydroxyphenyl)chromone | 59.71452747 | 286.25 | White Mulbrry Root-bark |
| MOL002514 | Sexangularetin | 62.85791915 | 316.28 | White Mulbrry Root-bark |
| MOL000554 | gallic acid-3-O-(6'-O-galloyl)-glucoside | 30.25032187 | 484.4 | White Mulbrry Root-bark |
| MOL009653 | Cycloeucalenol | 39.72647216 | 426.8 | White Mulbrry Root-bark |
| MOL000228 | (2R)-7-hydroxy-5-methoxy-2-phenylchroman-4-one | 55.23317389 | 270.3 | Baikal Skullcap Root |
| MOL002714 | baicalein | 33.51891869 | 270.25 | Baikal Skullcap Root |
| MOL002908 | 5,8,2'-Trihydroxy-7-methoxyflavone | 37.00837363 | 300.28 | Baikal Skullcap Root |
| MOL002909 | 5,7,2,5-tetrahydroxy-8,6-dimethoxyflavone | 33.81582599 | 376.34 | Baikal Skullcap Root |
| MOL002910 | Carthamidin | 41.15096273 | 288.27 | Baikal Skullcap Root |
| MOL002911 | 2,6,2',4'-tetrahydroxy-6'-methoxychaleone | 69.03987557 | 302.3 | Baikal Skullcap Root |
| MOL002913 | Dihydrobaicalin_qt | 40.03778103 | 272.27 | Baikal Skullcap Root |
| MOL002914 | Eriodyctiol (flavanone) | 41.35042713 | 288.27 | Baikal Skullcap Root |
| MOL002915 | Salvigenin | 49.06592606 | 328.34 | Baikal Skullcap Root |
| MOL002917 | 5,2',6'-Trihydroxy-7,8-dimethoxyflavone | 45.04742802 | 330.31 | Baikal Skullcap Root |
| MOL002925 | 5,7,2',6'-Tetrahydroxyflavone | 37.01348688 | 286.25 | Baikal Skullcap Root |
| MOL002926 | dihydrooroxylin A | 38.71506565 | 286.3 | Baikal Skullcap Root |
| MOL002927 | Skullcapflavone II | 69.51043398 | 374.37 | Baikal Skullcap Root |
| MOL002928 | oroxylin a | 41.367569 | 284.28 | Baikal Skullcap Root |
| MOL002932 | Panicolin | 76.25704989 | 314.31 | Baikal Skullcap Root |
| MOL002933 | 5,7,4'-Trihydroxy-8-methoxyflavone | 36.56200469 | 300.28 | Baikal Skullcap Root |
| MOL002934 | NEOBAICALEIN | 104.3446052 | 374.37 | Baikal Skullcap Root |
| MOL002937 | DIHYDROOROXYLIN | 66.06173872 | 286.3 | Baikal Skullcap Root |
| MOL000525 | Norwogonin | 39.40397184 | 270.25 | Baikal Skullcap Root |
| MOL000552 | 5,2'-Dihydroxy-6,7,8-trimethoxyflavone | 31.71246493 | 344.34 | Baikal Skullcap Root |
| MOL000073 | ent-Epicatechin | 48.95984114 | 290.29 | Baikal Skullcap Root |
| MOL001458 | coptisine | 30.671852 | 320.34 | Baikal Skullcap Root |
| MOL001490 | bis[(2S)-2-ethylhexyl] benzene-1,2-dicarboxylate | 43.59332547 | 390.62 | Baikal Skullcap Root |
| MOL002879 | Diop | 43.59332547 | 390.62 | Baikal Skullcap Root |
| MOL002897 | epiberberine | 43.09233228 | 336.39 | Baikal Skullcap Root |
| MOL008206 | Moslosooflavone | 44.08795959 | 298.31 | Baikal Skullcap Root |
| MOL010415 | 11,13-Eicosadienoic acid, methyl ester | 39.27534422 | 322.59 | Baikal Skullcap Root |
| MOL012245 | 5,7,4'-trihydroxy-6-methoxyflavanone | 36.62688628 | 302.3 | Baikal Skullcap Root |
| MOL012246 | 5,7,4'-trihydroxy-8-methoxyflavanone | 74.23522001 | 302.3 | Baikal Skullcap Root |
| MOL012266 | rivularin | 37.94023355 | 344.34 | Baikal Skullcap Root |
