# Supplementary figures and images for: Xuanfei Formula inhibited RSV infection by normalizing the SREBP2-mediated cholesterol synthesis process
Source: Front Microbiol. 2024 May 2;15:1387062. doi: 10.3389/fmicb.2024.1387062 (PMC11100329; doi:10.3389/fmicb.2024.1387062)

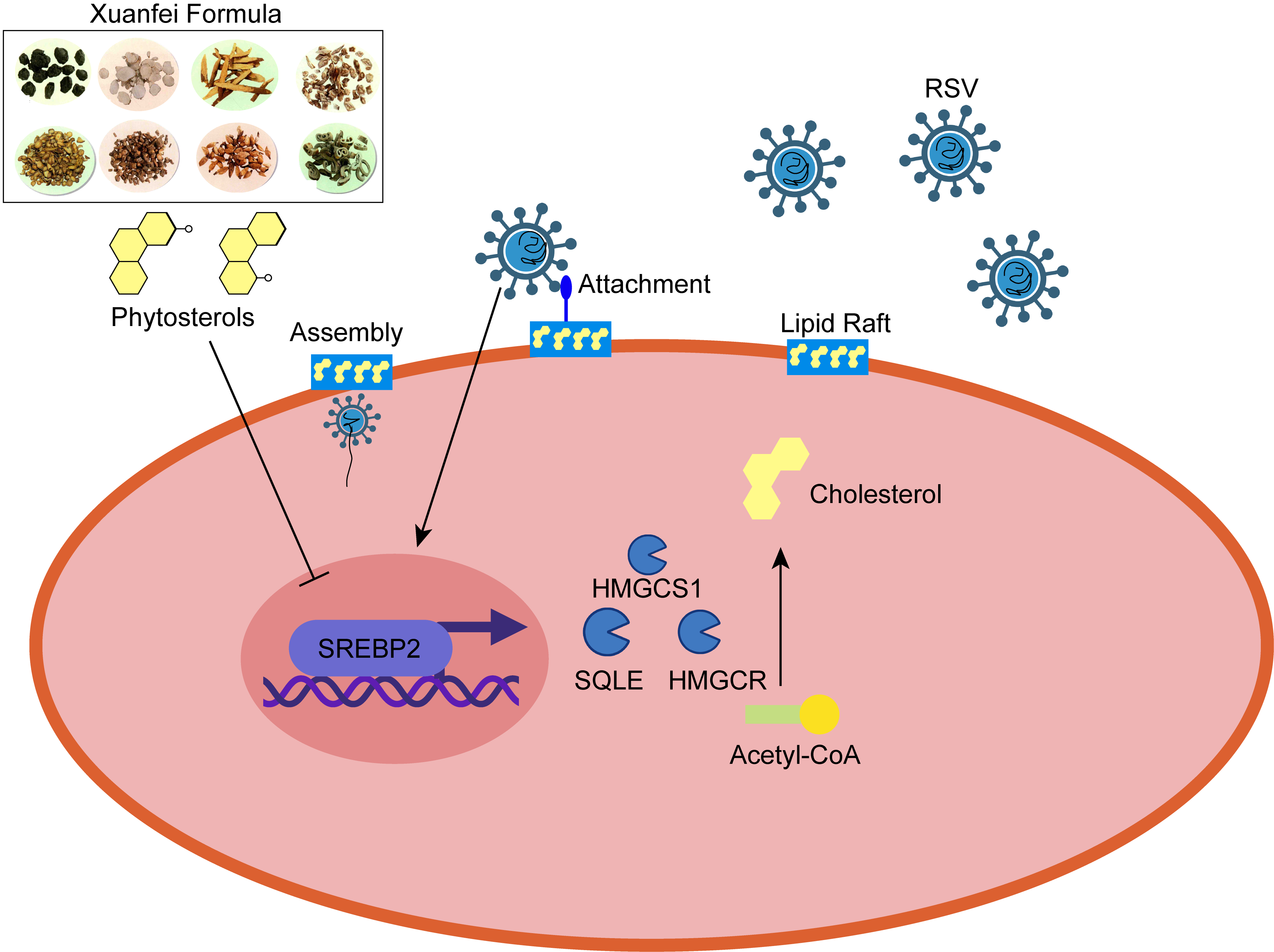

Supplement: Supplementary file 2 [file Image_1.TIF]
